# Insights into human eNOS, nNOS and iNOS structures and medicinal indications from statistical analyses of their interactions with bound compounds

**DOI:** 10.52601/bpr.2023.210045

**Published:** 2023-06-30

**Authors:** Jianshu Dong, Dié Li, Lei Kang, Chenbing Luo, Jiangyun Wang

**Affiliations:** 1 School of Pharmaceutical Sciences, Zhengzhou University, Zhengzhou 450001, China; 2 Institute of Drug Discovery and Development, Zhengzhou University, Zhengzhou 450001, China; 3 Key Laboratory of Advanced Drug Preparation Technologies, Ministry of Education of China, Zhengzhou University, Zhengzhou 450001, China; 4 Collaborative Innovation Center of New Drug Research and Safety Evaluation, Henan Province, Zhengzhou University, Zhengzhou 450001, China; 5 Key Laboratory of Henan Province for Drug Quality control and Evaluation, Zhengzhou University, Zhengzhou 450001, China; 6 Laboratory of RNA Biology, Institute of Biophysics, Chinese Academy of Sciences, Beijing 100101, China

**Keywords:** Nitric oxide synthase, Endothelial NOS, Neuronal NOS, Drug design, Molecular pharmacology, Inducible NOS

## Abstract

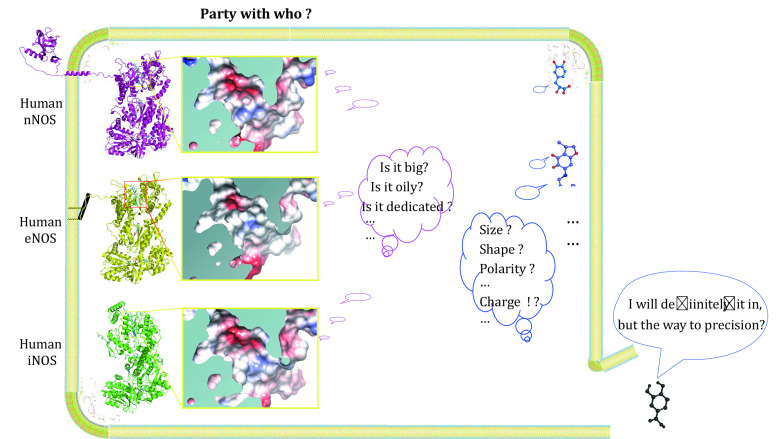

83 Structures of human nNOS, 55 structures of human eNOS, 13 structures of iNOS, and about 126 reported NOS-bound compounds are summarized and analyzed. Structural and statistical analysis show that, at least one copy of each analyzed compound binds to the active site (the substrate arginine binding site) of human NOS. And binding features of the three isoforms show differences, but the binding preference of compounds is not in the way helpful for inhibitor design targeting nNOS and iNOS, or for activator design targeting eNOS. This research shows that there is a strong structural and functional similarity between oxygenase domains of human NOS isoforms, especially the architecture, residue composition, size, shape, and distribution profile of hydrophobicity, polarity and charge of the active site. The selectivity and efficacy of inhibitors over the rest of isoforms rely a lot on chance and randomness. Further increase of selectivity via rational improvement is uncertain, unpredictable and unreliable, therefore, to achieve high selectivity through targeting this site is complicated and requires combinative investigation. After analysis on the current two targeting sites in NOS, the highly conserved arginine binding pocket and H4B binding pocket, new potential drug-targeting sites are proposed based on structure and sequence profiling. This comprehensive analysis on the structure and interaction profiles of human NOS and bound compounds provides fresh insights for drug discovery and pharmacological research, and the new discovery here is practically applied to guide protein-structure based drug discovery.

## INTRODUCTION

Nitric oxide (NO) is an important gaseous small molecule involved in signaling transduction (Murad *et al.* 1993), immune response, blood vessel dilation, endocrine function and many other fundamental physiological processes in the nervous, immune and cardiovascular systems (Moncada and Higgs 1991; Murad *et al.* 1993). In the human body, NO is mainly produced by nitric oxide synthase (NOS) (Bian and Murad 2003). Endothelial NOS (eNOS or NOS3), neuronal NOS (nNOS or NOS1) and inducible NOS (iNOS or NOS2) are the three NOS isoforms that human bodies produce (Cinelli *et al.* 2020a; Daiber *et al.* 2019; Li and Poulos 2005; Nathan and Xie 1994). In mammals, both eNOS and nNOS are constitutively expressed (Nathan and Xie 1994).

**Figure 1:**
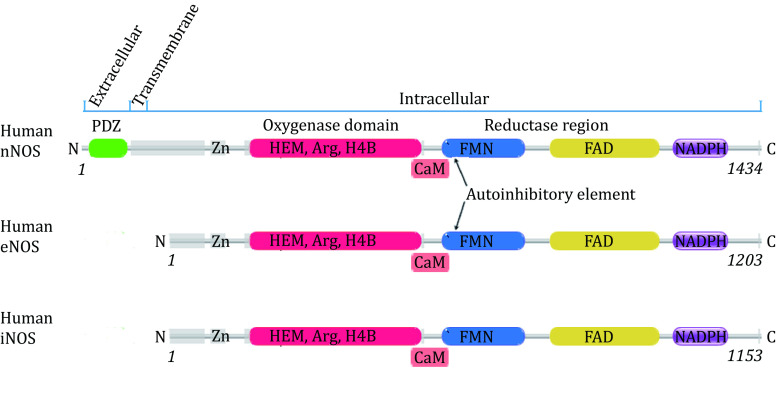
Organisation of human NOS domains. The annotation is based on polypeptide sequence of human nNOS, eNOS and iNOS. Human iNOS and human eNOS have very similar domain organisations from the N terminal to the C terminal. Human nNOS has an additional extracellular N terminal PDZ domain (shown in green) and a transmembrane helix compared to eNOS and iNOS. Zinc binding sites, oxygenase domain with HEM, substrate L-Arginine and H4B binding sites, reductase region with FMN, FAD and NADPH binding sites of these three human NOS isoforms are presented. Lipid modification of human eNOS can occur (palmitoylation at Cys15 and Cys26, and Myristoylation at Gly2) that facilitates its anchor onto the membrane. Both Human nNOS and eNOS have auto-inhibitory elements which human iNOS lacks. Regulatory Calmodulin is shown by “CaM” near the CaM binding sites

With 50%–59% mutual overall polypeptide sequence identity between human eNOS, iNOS and nNOS, the three isoforms are highly similar to each other in several ways (Fig. 1); they carry the same cofactors, utilize the same substrate and catalyze the same reaction to generate the same product nitric oxide (Griffith and Stuehr 1995; Hemmens and Mayer 1998; Stuehr and Haque 2019). All three human NOS isoforms have a zinc-binding motif and heme domain near the N-terminal (Fischmann *et al.* 1999; Raman *et al.* 1998), a flavin mononucleotide (FMN)-containing flavodoxin region, a flavin adenine dinucleotide (FAD)-bearing region, and a nicotinamide adenine dinucleotide phosphate (NADPH)-binding region in the C-terminal that can function as a reductase (Fig. 1) (Hall *et al.* 1994; Hemmens and Mayer 1998; Stuehr and Haque 2019). Human NOS are heme b-containing proteins (Crane *et al.* 1997; Raman *et al.* 1998). The heme domain (or oxygenase domain) is also responsible for tetrahydrobiopterin (H4B) and substrate L-arginine binding (Fischmann *et al.* 1999; Raman *et al.* 1998).

In the presence of NADPH, proton, H4B and dioxygen, NOS catalyzes the generation of NO from natural substrate L-arginine (Adak *et al.* 2000; Wei *et al.* 2005). Electrons abstracted from the arginine transfer between NADPH, FAD, FMN, and the active site heme b (Griffith and Stuehr 1995). Dioxygen is the ultimate electron acceptor. NO and L-citrulline are produced after arginine oxidation (Hemmens and Mayer 1998). The reaction catalyzed by human NOS is as follows:

3H^+ ^ + 2L-Arginine + 3NADPH + 4O_2_ $ \xrightarrow{\;\;\;\;\;\;\;\;\;\;}$ 4H_2_O + 2L-citrulline + 3NADP^+ ^ + 2NO

The catalytic activity of all three NOS isoforms requires dimerization of the protein via the heme domain. H4B cofactor is essentially required for the proper functionality of human NOS (Adak *et al.* 2000; Lajoix *et al.* 2004; Wei *et al.* 2005). And like many other cofactor-containing proteins (Bushmarina *et al.* 2006; Curnow and Booth 2010), the presence of H4B increases the stability of the NOS protein (Rodriguez-Crespo *et al.* 1997). The alpha helix between the heme domain and FMN-binding region is responsible for calmodulin (CaM, calcium-modulated protein) binding (Aoyagi *et al.* 2003; Cinelli *et al.* 2020a; Li and Poulos 2005; Piazza *et al.* 2016). Calmodulin is a regulatory protein of human NOS and can be activated by the increase of cytoplasmic calcium level (Nathan and Xie 1994). The calmodulin-binding position is also conserved among three NOS isoforms (Fig. 1), although the binding of human iNOS to CaM is irreversible, while nNOS and eNOS bind CaM reversibly in response to intracellular Ca^2+ ^ concentration (Nathan and Xie 1994).

The differences between the three human NOS isoforms are significant. Human nNOS has an additional extracellular N terminal PDZ domain and a transmembrane region compared to eNOS and iNOS (Fig. 1) (Hillier *et al.* 1999). The human eNOS is a water-soluble peripheral membrane protein that can anchor to and associate with the membrane of the cell after co-translational myristoylation at Gly2 or post-translational palmitoylation at Cys15 and Cys26 (Dudzinski *et al.* 2006; GarciaCardena *et al.* 1996; Nakane *et al.* 1991; Wei *et al.* 2011). The lipid modiﬁcation state affects the localization and activity of human eNOS. Human iNOS is cytosolic, and no lipid modification or association with the membrane has been reported. Both Human nNOS and eNOS have auto-inhibitory elements that human iNOS lacks (Nishida and de Montellano 1999, 2001).

All three isoforms of NOS are closely linked to human health. Excess NO from either nNOS or iNOS is directly implicated in human disorders as discussed below. The excess NO from nNOS in tissues of the peripheral and central nervous system is directly linked to disorders including motor neuron disease, neurodegenerative diseases such as Parkinson’s, Huntington’s, Alzheimer’s disease, and amyotrophic lateral sclerosis, *etc*. (Tripathi *et al.* 2020; Uehara *et al.* 2006).

The Ca^2+^-insensitive iNOS can be induced by cytokines or pathogens, and it produces NO to kill bacteria, microbial-infected cells and tumor cells. Overproduction of NO by iNOS has been implicated in inflammation, rheumatoid arthritis, infection susceptibilities, inflammatory bowel disease, immune-type diabetes, stroke, sepsis, thrombosis, cancer (Bian and Murad 2003; Thippeswamy *et al.* 2006) and multiple sclerosis (Duncan and Heales 2005). And inhibition of iNOS decreases cellular senescence (Qiao *et al.* 2022). Therefore, overexpression, hyperactivity, or dysregulation of iNOS leads to various human diseases and pain (Cinelli *et al.* 2020a).

Malfunction or dysregulation of eNOS in both human and animal models are related to disorders including oxidative stress, endothelial dysfunction, vascular disease, diabetic retinopathy, *etc*. (Bian and Murad 2003; Daiber *et al.* 2019; Moncada and Higgs 1991). eNOS plays an important role in the cardiovascular systems through the regulation of the diameter of blood vessels and maintenance of an antiproliferative and anti-apoptotic environment (Dudzinski *et al.* 2006; Forstermann and Munzel 2006). Mutation of eNOS Glu298 to aspartate is probably associated with susceptibility to coronary spasm (Yoshimura *et al.* 1998). In addition, increased expression or elevated protein level of eNOS has vasodilatory, anti-inflammatory, antithrombotic, antiproliferative and cardiovascular protective effects (Daiber *et al.* 2019). It has been shown that eNOS may be related to mitochondrial biogenesis and energy homeostasis of the cell as well (Le Gouill *et al.* 2007). Polycyclic aromatic hydrocarbon pollutants may harm the human body through abnormal inhibition of eNOS (Wu *et al.* 2020). Therefore, it is generally recognized nowadays that human eNOS protein cannot be inhibited while the similarly important homologous protein iNOS is targeted by drugs for endotoxemia, inflammatory, neuropathic pain, and arthritis treatment, *etc.* (Cinelli *et al.* 2020a; Garcin *et al.* 2008), or nNOS is targeted by drugs for neurodegenerative disorders (Cinelli *et al.* 2020b), *etc*.

To increase the selectivity of iNOS and nNOS inhibitors or eNOS interactors is one of the most urgent and important issues. So far, there are no inhibitors of iNOS or nNOS, or compounds that selectively bind to eNOS, that have been approved for clinical use in humans (Janaszak-Jasiecka *et al.* 2023; Shi *et al.* 2023). And human eNOS protein, the attractive therapeutic target (Roe and Ren 2012), has not been medicinally or pharmacologically fully exploited yet. It has been widely recognized that eNOS activation, recoupling, and increased NO bioavailability are therapeutic strategies for a series of cardiac vascular diseases and diabetes, including hypertension, pulmonary hypertension, arterial stiffness and heart failure, *etc*. (Faria *et al.* 2012; Janaszak-Jasiecka *et al.* 2023; Roe and Ren 2012). Although the difficulty in developing stabilizers or activators is well known and well recognized, the lack of analysis on the interactions between available compounds and NOS definitely hinders the discovery of eNOS activators, stabilizers or enhancers. To further the understanding towards a clear relationship among compounds’ property, binding profile and effect on the NOS enzyme, and to promote the design of isoform-selective NOS interactors to treat inflammation, arthritis, stroke, septic shock, neurodegenerative disorders, cardiovascular disorders and cancer, a systematic analysis on the available structures of human eNOS, nNOS and iNOS, is carried out in this study.

## RESULTS

There are now about 55 human eNOS, 83 human nNOS and 13 human iNOS structures deposited in the Protein Data Bank in total, and these structures are mainly resolved by using the X-ray crystallography method. There are four deposited structures of short eNOS peptide (around Ala500) and two structures of iNOS peptide (510–531) complexed with calmodulin that are determined by NMR (eNOS (1NIW, 2LL7, 2MG5, 2N8J), iNOS (2LL6, 5TP6)), and another short eNOS peptide Gly30–Pro36 complexed with PILR alpha immune cell receptor (5XOF), and short iNOS peptide ~Lys22–Glu29 complexed with SPSB2 (5XN3, 6JWM, 6JWN, 6KEY), and all these eleven depositions are not included in statistical analysis. These structures have no inhibitor bound. The 83 human nNOS structures and the remaining 51 eNOS and the iNOS structures are that of a truncated protein, containing only N-terminal heme domain (nNOS (~Cys302–Lys722), eNOS (~Ala41/Lys67–Trp480/Lys481)), with molecular weight of the dimer about 95 kDa. The resolution of these 51 eNOS structures ranges from 1.73 to 2.56 Å, and careful inspection of these 51 depositions reveals good quality of both diffraction data and built molecular model (supplementary Table S1). By using UCSF Chimera, structures of eNOS are superimposed, visualized and analyzed. Root mean square deviations (RMSD) of backbone atoms between any two of these monomer structures range from ~0.2 Å to about 1 Å, showing good confidence in the structural data (supplementary Table S1). When properly superimposed, the RMSD between heme b atoms from any two structures is below 0.2 Å. These reliable accurate experimental structures of human NOS become a gorgeous resource for three-dimensional statistical analysis.

### Characteristics of bound compounds

Both the size and hydrophobicity of inhibitors contribute to the energetically favored energy-releasing binding to eNOS. The binding features of compounds to nNOS, iNOS and eNOS do show differences.

**Figure 2:**
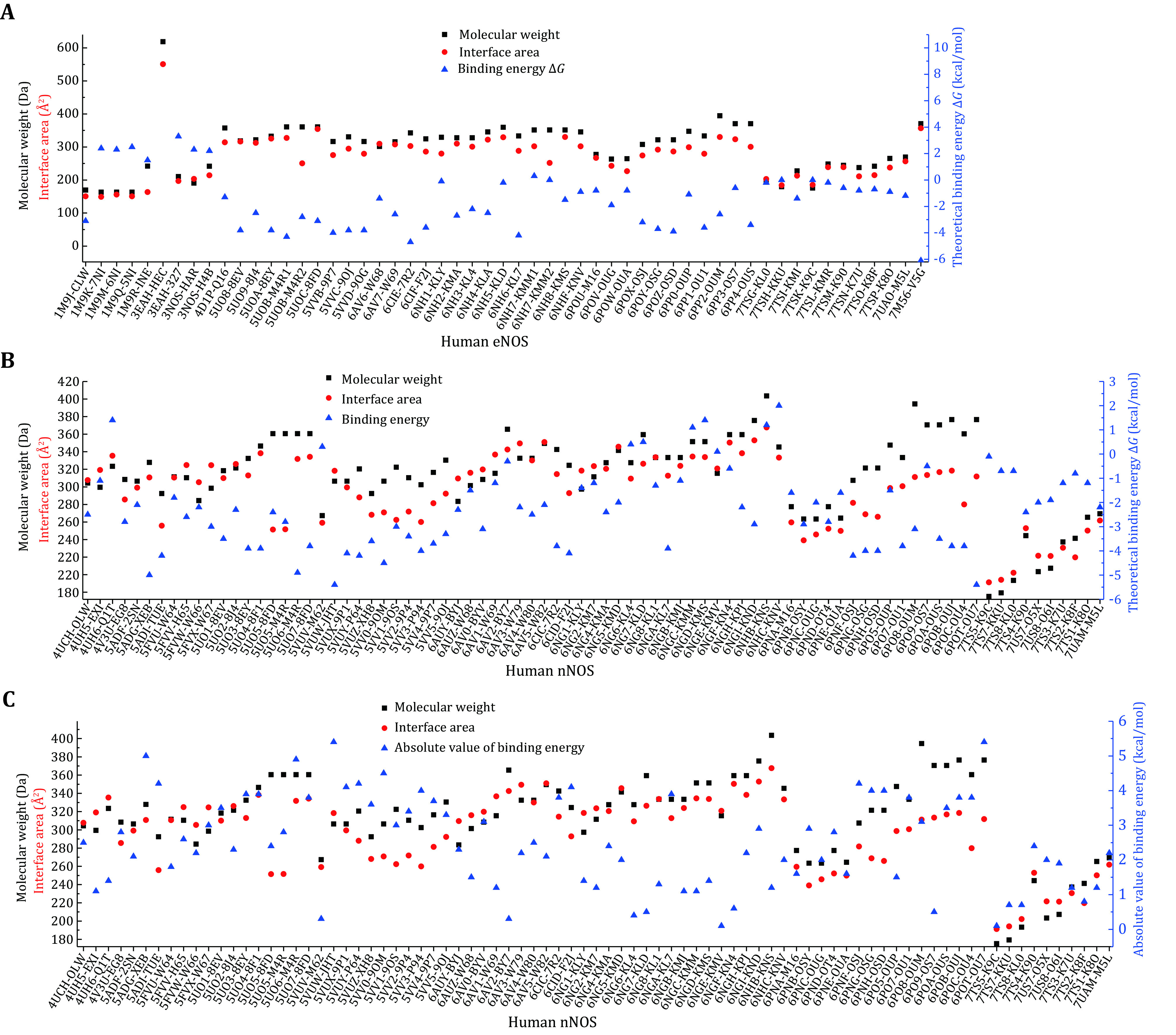
Scatter plot showing molecular weights, binding interface areas, and theoretical standard binding free energies (Δ*G*) of inhibitory compounds bound to human eNOS and nNOS. **A** Human eNOS. The binding interface area is positively correlated with molecular weight, while negative correlation dominates the relationship between theoretical binding energy value and binding interface area. The binding energy of HEC (an heme analogue, 3EAH.pdb) is omitted as it’s beyond the current scale. For 8EY (5UOA.pdb), M4R (5UOB.pdb), KMM (6NH7.pdb), KL4 (6NH3.pdb) and M16 (6POU.pdb), only one copy of the compound molecule which is in the active site is included. **B** Human nNOS. Neither binding interface area nor molecular weight is correlated to the value of theoretical binding energy in a consistent fashion. **C** Binding interface area is not consistently positively correlated to the absolute value of theoretical binding energy for human nNOS

The molecular weight of the compound, binding interface area and theoretical binding energy of different compounds to human eNOS, iNOS, and nNOS are summarized. As expected, a positive correlation between binding interface area and molecular weight can be observed in eNOS-compounds interactions (Fig. 2, supplementary Table S2). Because the inhibitors discussed here are mostly composed of common abundant elements in living organisms, molecular weight directly indicates the size of the compound. This figure means that the larger the compound, the larger the binding interface area. The relationship between theoretical binding free energy (Δ*G* value) and binding interface area (or molecular weight) of the compound is complicated, and negative correlation is the case for a lot of compound-eNOS interactions (Fig. 2A). For the majority of eNOS bound compounds, the larger the interface area, the more energetically favored binding (supplementary Table S2). The phenomenon is consistent with the common sense that the overall strength of weak interactions relies heavily on interface area, and weak interactions between certain atoms with certain geometry are simply stronger than others. However, no similar systematic abstraction can be summarized from the compound-nNOS interactions (Fig. 2B and 2C, supplementary Table S3). To our surprise, statistically, the absolute value of theoretical binding energy is not consistently positively correlated to the binding interface area, for either eNOS (supplementary Table S2 and Fig. S1) or nNOS (Fig. 2C, supplementary Table S3).

**Figure 3:**
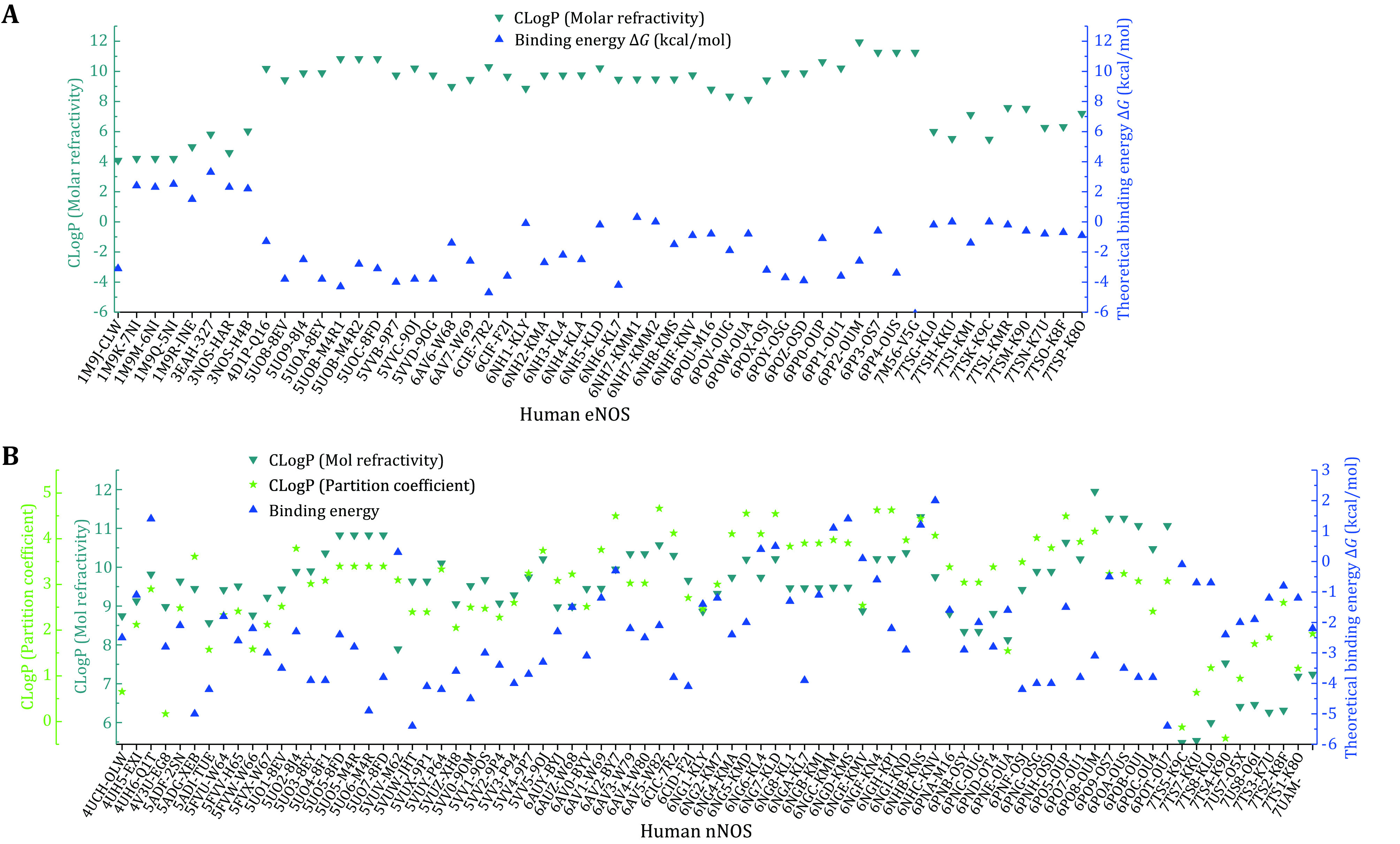
Plot of ClogPs (molar refractivity, partition coefficient) and theoretical binding energies of human eNOS- and nNOS-bound compounds. **A** For eNOS, the theoretical binding energy value is negatively correlated with hydrophobicity or lipophilicity (represented by theoretical CLogP values) of the compound, which means lipophilicity constructively contributes to the energetically favored heat-releasing binding to eNOS. **B** No such pattern is observed for human nNOS

The hydrophobicity and hydrophilicity properties of the bound compounds are then analyzed. LogP indicates the hydrophobicity or lipophilicity of the compound, and the theoretical CLogP value of each compound is worked out by using both the partition coefficient and the molar refractivity approach (supplementary Tables S2 and S3). ClogP of the compound and the theoretical binding free energy Δ*G* are then plotted against compounds (Fig. 3). Negative correlation between Δ*G* value and molar refractivity ClogP suggests that hydrophobic compounds are probably much more energetically favored by the eNOS binding pocket (Fig. 3A). From Fig. 2 and Fig. 3, it can be inferred that, statistically, both binding interface area and hydrophobicity (or lipophilicity) of the compound constructively contribute to the energetically favored affinity between human eNOS protein and compounds. But no such pattern is observed for human nNOS (Fig. 2B and 2C, Fig. 3B) or iNOS.

### Attributes of the active sites of human eNOS, nNOS and iNOS

The binding of compounds to human NOS active site prohibits binding of substrate arginine, and the shape, size, hydrophobicity, polarity, charge and residue composition of this pocket, are highly similar among NOS isoforms, with the dissimilarity not helpful for nNOS and iNOS inhibitor design to let go of eNOS.

**Figure 4:**
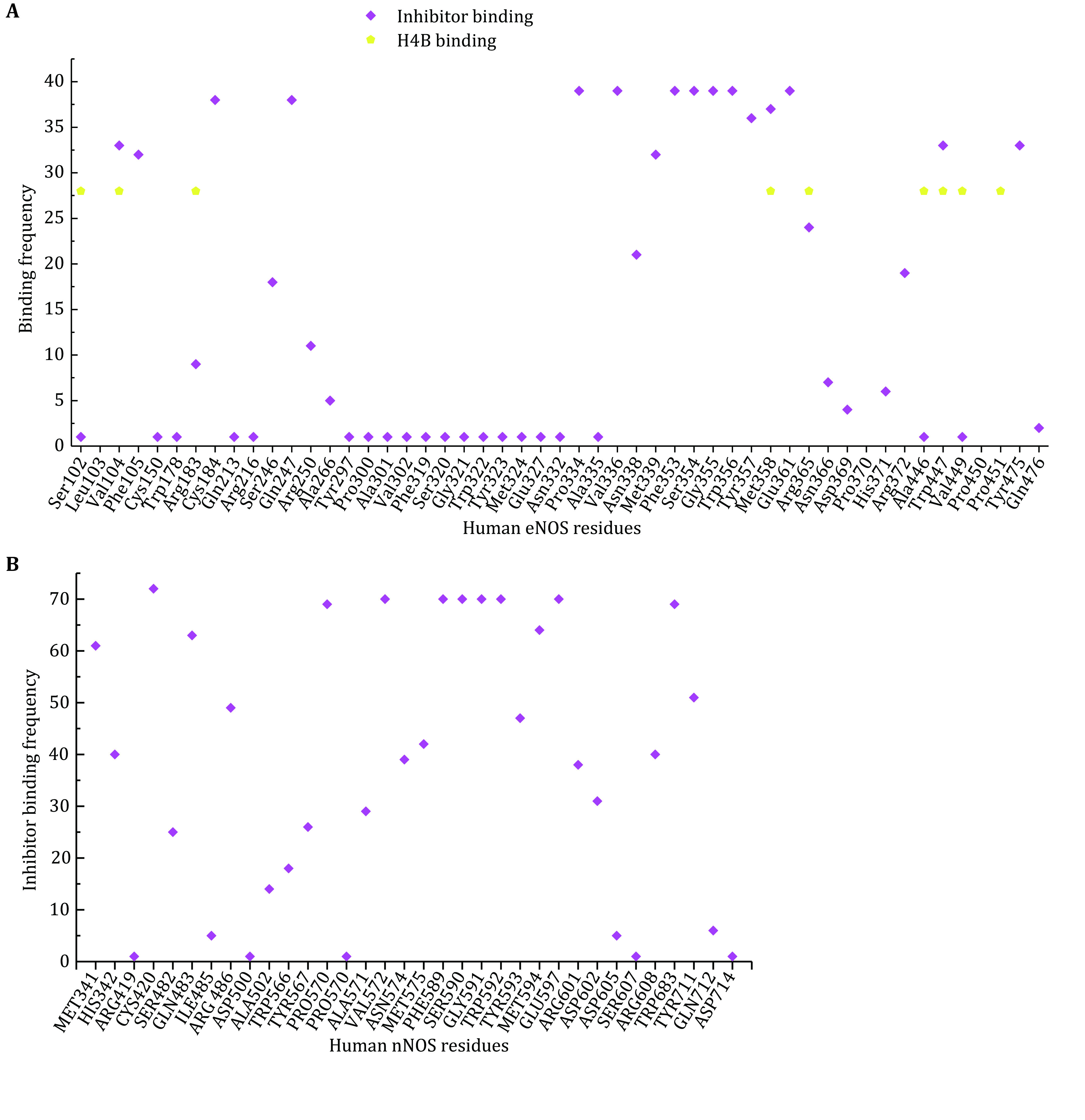
Statistical summary of the interaction frequency of human NOS residues with inhibitors or H4B. *X*-axis is amino acid residues of human NOS, and *Y*-axis is the involvement frequency of the residue in compound binding. **A** Human eNOS. Although there are residues which interact with both inhibitors and H4B, like Val104 and Arg365, distinct binding regions of inhibitors and H4B can be observed. This is consistent with Fig. 5. The binding frequencies of residues to H4B are normalized to 28 with a threshold of 15. **B** Human nNOS

**Figure 5:**
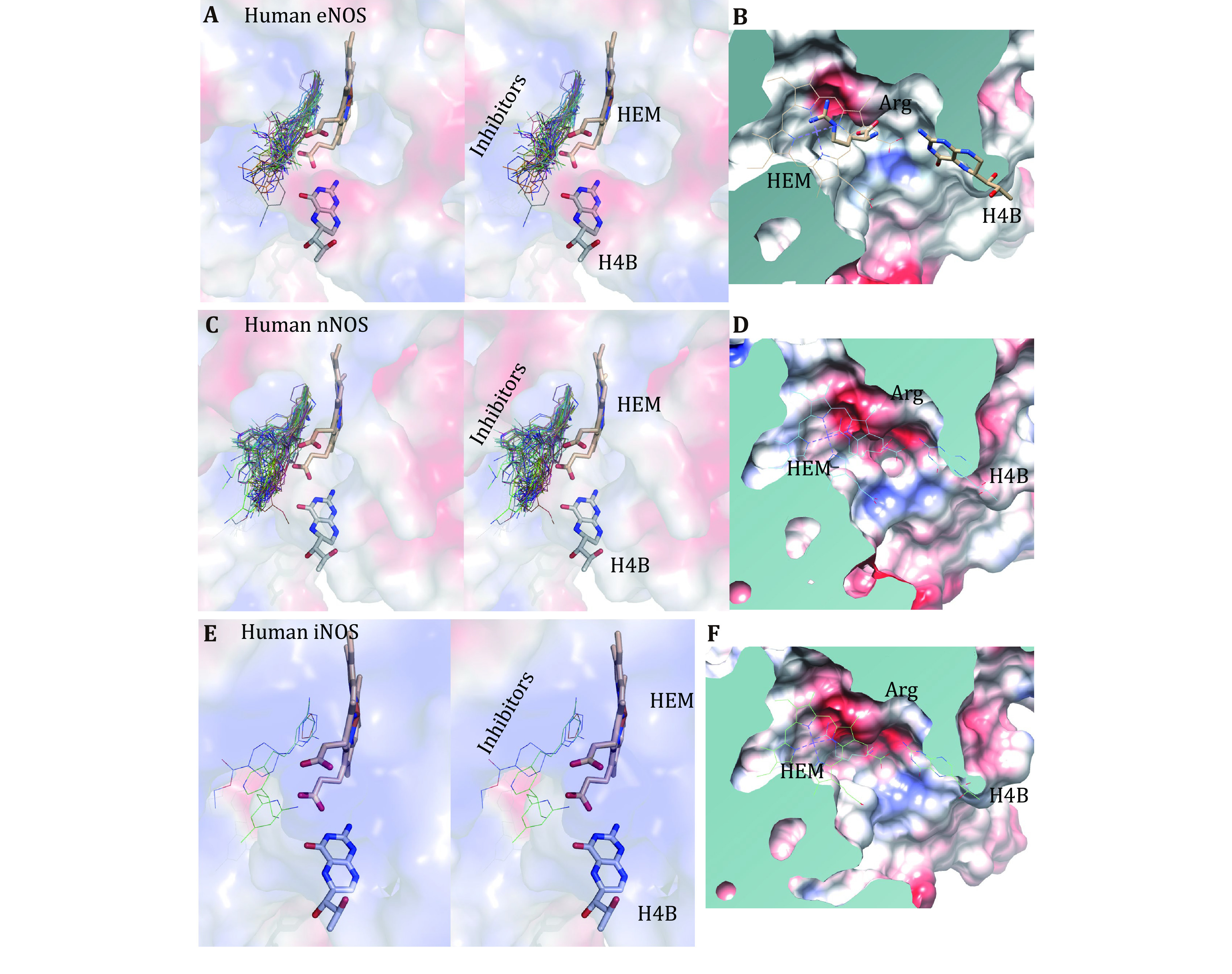
Binding profiles of inhibitors, H4B, HEM and substrate arginine to human eNOS, human nNOS and human iNOS. **A** Stereo view of the superposed active site of eNOS structures with inhibitors shown in thin lines, and H4B and HEM shown in sticks. **B** Surface capping views of the human eNOS active site. HEM is shown in thin lines; H4B and substrate arginine are shown in sticks. The slight hydrophobicity of both the arginine binding site and the H4B binding site can be observed, which is consistent with Fig. 3A and Fig. 6. The inhibitor binds to the substrate arginine-binding pocket, and this is far away from the H4B binding site. **C** Stereo view of the superposed active site of nNOS structures with inhibitors shown in thin lines, and H4B and HEM shown in sticks. **D** Surface capping views of the human nNOS active site. HEM, H4B and substrate arginine are shown in thin lines. The charge distribution and hydrophobicity (or hydrophilicity) properties of both the arginine-binding site and the H4B-binding site are similar to that of human eNOS. **E** Stereo view of the superposed active site of iNOS structures with inhibitors shown in thin lines, and H4B and HEM shown in sticks. **F** Surface capping views of the human iNOS active site (1NSI.pdb)

The interaction profiles between compound and human eNOS, nNOS and iNOS, are analyzed in detail. For each of the 51 human eNOS structures and 83 human nNOS structures, the interface residues involved in compound binding are extracted, summarized and analyzed (Fig. 4, supplementary Table S4 and S5). The contribution of each residue to the theoretical binding energy is then analyzed. The binding region of those inhibitors is found to be highly conserved (Fig. 5, supplementary Table S4 and S5). One copy of the following five compound molecules (8EY (5UOA.pdb, 5UO3.pdb), M4R (5UOB.pdb), KMM (6NH7.pdb), KL4 (6NH3.pdb) and M16 (6POU.pdb)), and all other inhibitors bind to the pocket near the heme moiety. This region is actually the substrate arginine binding site (catalytic center) of the human NOS protein (Fig. 5).

The substrate arginine-binding pocket of NOS is further analyzed; it is deep, and it’s modest in size. The compounds interact with both heme and polypeptide chains. After superimposing published human eNOS (Fig. 5A), human nNOS (Fig. 5C) and human iNOS (Fig. 5E) structures, the active sites are rendered in stereo views, inhibitors are shown in thin lines, and H4B and HEM are shown in sticks (Fig. 5A, 5C and 5E). Then the three-dimensional binding profiles of inhibitors, H4B, HEM and substrate arginine to human eNOS, human nNOS and human iNOS are analyzed in detail (Fig. 5). It is clear that the substrate arginine-binding pocket is different from H4B binding site. Consistent with Fig. 4, the analyzed compounds are targeting the highly conserved substrate arginine-binding pocket of human NOS.

**Figure 6:**
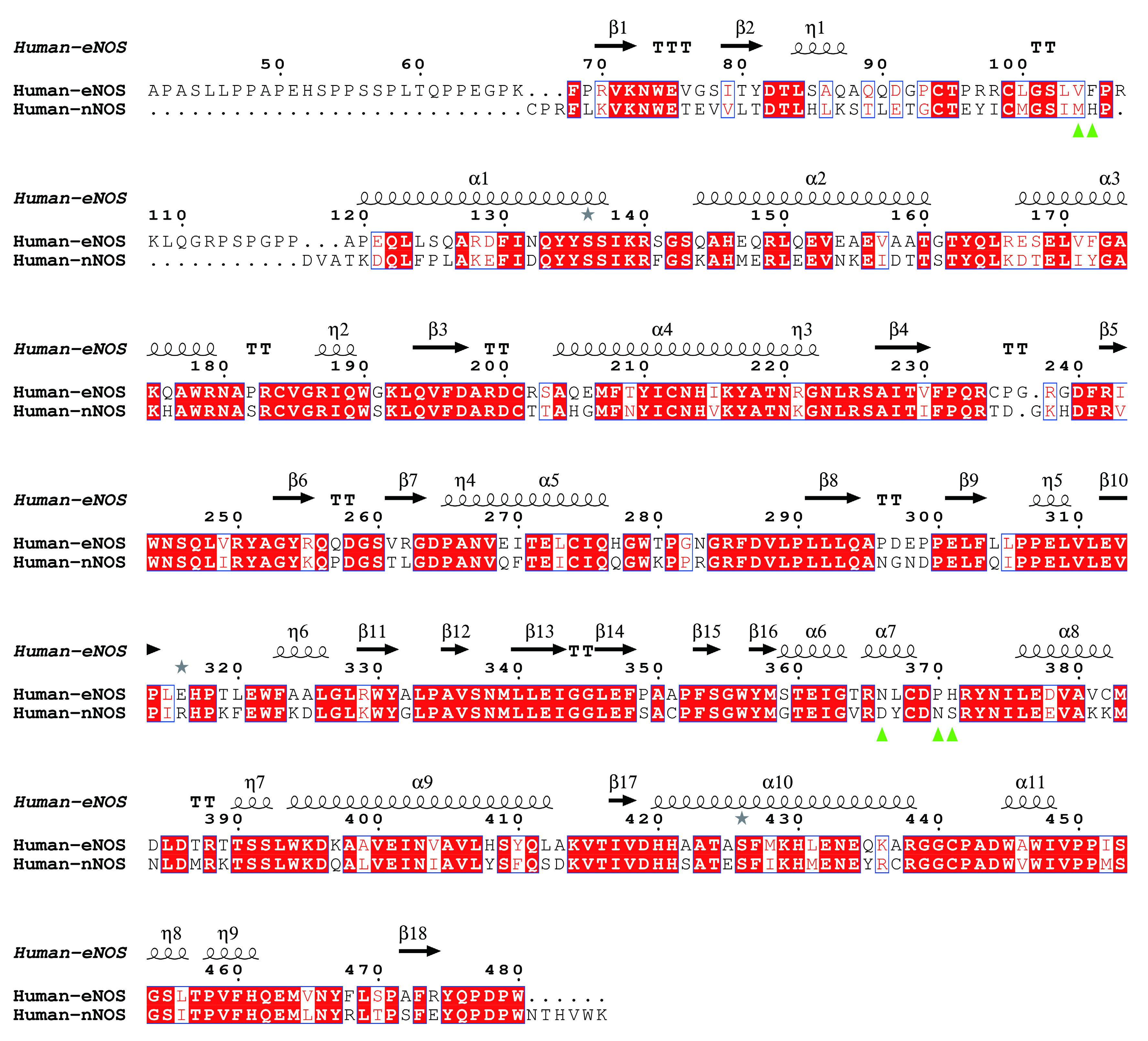
Sequence alignment of human eNOS and human nNOS based on structure superimposition. eNOS monomer from 4d1o.pdb and nNOS monomer from 6av3.pdb are superposed and aligned by using UCSF Chimera (Pettersen *et al.* 2004) and ESPript (Robert and Gouet 2014). Human eNOS residues Val104, Phe105, Asn366, Pro370, His371, which corresponds to human nNOS Met341, His342, Asp602, Asn606, Ser607, are highlighted by green triangles. Among these residues, eNOS-Asn366 and nNOS-Asp602 are within the substrate arginine binding pocket, and Val104, Phe105, Pro370, His371, Met341, His342, Asn606, Ser607 are located at the opening of the cavity. RMSD between human eNOS (4d1o.pdb) and human nNOS (6av3.pdb) monomers is ~0.61 Å

**Figure 7:**
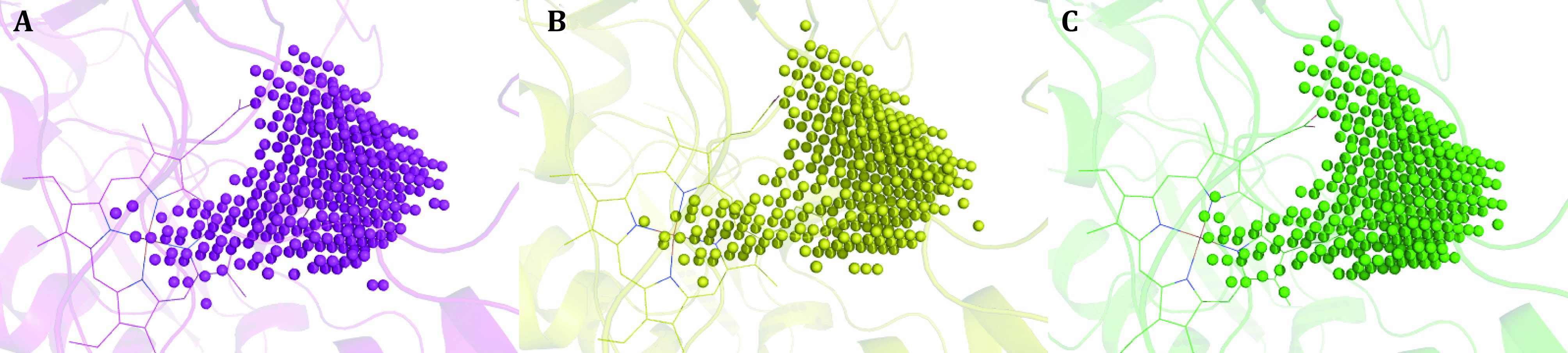
Dummy atom filled models of human NOS catalytic pockets (substrate arginine binding pocket). The upper right corner of each panel is the mouth of the pocket, and heme b is shown in thin lines. **A** Human nNOS (6po8.pdb). **B** Human eNOS (5uo9.pdb). **C** Human iNOS (4cx7.pdb)

To get a better understanding of the characters of this contemporary hot spot of drug discovery, the electrostatic property of the binding pockets of human eNOS, nNOS, and iNOS are worked out and compared; surface capping views of the electrostatic surface of human NOS active sites are displayed, human eNOS (Fig. 5B), human nNOS (Fig. 5D) and human iNOS (Fig. 5F). HEM is shown in thin lines; H4B and substrate arginine are shown in sticks (Fig. 5B). Because the binding orientation of HEM, H4B and arginine to the three isoforms are virtually the same, they are shown in thin lines for human nNOS (Fig. 5D) and human iNOS (Fig. 5F). The slight hydrophobicity of both arginine binding site and H4B binding site of human eNOS can be observed (Fig. 5B), which is consistent with Fig. 3A and Fig. 6. The charge distribution and hydrophobicity (or hydrophilicity) property of both arginine-binding site and H4B-binding site of human nNOS (Fig. 5D) and human iNOS (Fig. 5F) are similar to that of human eNOS. The similarities in charge and polarity distribution between these three human NOS isoforms (Fig. 5) are significant, but the differences are unignorable as well. The charged area of human eNOS is relatively smaller than nNOS and iNOS, which is consistent with Fig. 3 and the supplementary Tables S2 and S3. And it explains the different features in compound binding between human eNOS and nNOS observed in Fig. 3, this is probably because of the slight difference in hydrophobicity of their substrate arginine binding sites. Structure-based sequence alignment also supports this explanation (Fig. 6), more residues with hydrophobic side chains are present at the substrate binding site in human eNOS than nNOS (Residues Val104, Phe105, Asn366, Pro370, His371 of eNOS corresponding to Met341, His342, Asp602, Asn606, Ser607 of nNOS in structure superposition). The volume and the shape of substrate binding pockets of human NOS isoforms are also worked out by filling in with dummy atoms (Fig. 7, supplementary Structures 1–3), and the volumes of nNOS (6po8.pdb), eNOS (5uo9.pdb), and iNOS (4cx7.pdb) are 316, 322 and 321 Å^3^, respectively. The size (or volume), shape, and distribution pattern of hydrophobicity, polarity, and charge of the substrate binding pockets of human NOS isoforms are highly similar. Whether the similarity or the differences dominate is still to be discovered, and the unhelpfulness of the dissimilarity of this pocket for drug design will be further explained in the discussion section. There is not enough space in the active site pocket of NOS for the simultaneous binding of arginine and those analyzed inhibitors (Yu *et al.* 2010). The binding of these compounds to human NOS probably prohibits the binding of substrate arginine.

At least one copy of all analyzed inhibitors binds to the catalytic center of human NOS; and for some inhibitors (8EY, M4R, KMM, KL4 and M16), one additional copy binds to the H4B binding pocket of eNOS, and inhibitor 8EY also binds to the H4B binding pocket of human nNOS (5UO3.pdb). In this case, the inhibitor probably interferes with the binding of essential cofactor H4B as well.

### Mechanism of action

For the majority of human NOS-bound compounds, the inhibition effect is mainly through the prohibition of substrate arginine binding to the active site. H4B constructively contributes to the association between two NOS monomers via interaction with both. Disruption of H4B-NOS binding is the additional or alternative route of inhibition by some inhibitors.

Indeed, take eNOS (3NOS.pdb) for example, the binding interface areas between H4B and the two monomers are ~257 Å^2^ (with the contribution of heme included) and ~119 Å^2^, respectively, and with contributions of two H4B included, binding interface areas between the two monomers increase about 19% from ~2661 to ~3175 Å^2^. The two monomers of human NOS are held together mainly by non-covalent interactions. Non-covalent interactions, including hydrogen bonds, ionic interaction, van der Waals interactions, and hydrophobic effect, are weak individually, but can be strong collectively, which therefore strongly depends on the interface area. With the addition of H4B contributions, binding interface areas between the two nNOS and iNOS monomers increase from ~2683 to ~3147 Å^2^, and from ~2690 to ~3196 Å^2^, respectively (Table 1). The H4B binding site is distinct from the substrate binding pocket of NOS. The inhibition effect of these inhibitors (8EY, M4R, KMM, KL4 and M16) is probably a combined dual prohibition effect on substrate arginine binding and H4B cofactor binding.

**Table 1:** Interface areas between H4B and human NOS monomer, between H4B and HEM, and between two polypeptide chains from NOS dimers

Human NOS	Interface area between H4B and one monomer (Å^2^)	Interface area between H4B and the other monomer (Å^2^)	Interface area between H4B and HEM from one monomer (Å^2^)	Interface area between polypeptide chain from monomer A and monomer B (Å^2^)
eNOS	212	119	45	2661
nNOS	192	116	40	2683
iNOS	209	118	44	2690

## DISCUSSION

To summarize present NOS-inhibitor interactions, two binding sites are utilized by analyzed compounds in the inhibition of human NOS function, the substrate arginine-binding pocket (the active site) and the H4B-binding pocket. The binding of each of these inhibitors to the human NOS active site interferes with the binding of substrate arginine (Fig. 5), thus inhibiting the NO-generating activity. Based on the statistics here, it is clear that as long as a compound occupies the substrate-binding site, either fully or partially, the inhibition effect will dominate. Down to the bottom, this is the fundamental source of problems hindering selectivity. Although binding features of analyzed compounds to nNOS, iNOS and eNOS do show differences, no compound at either site has successfully achieved the required selectivity for clinical application yet.

**Figure 8:**
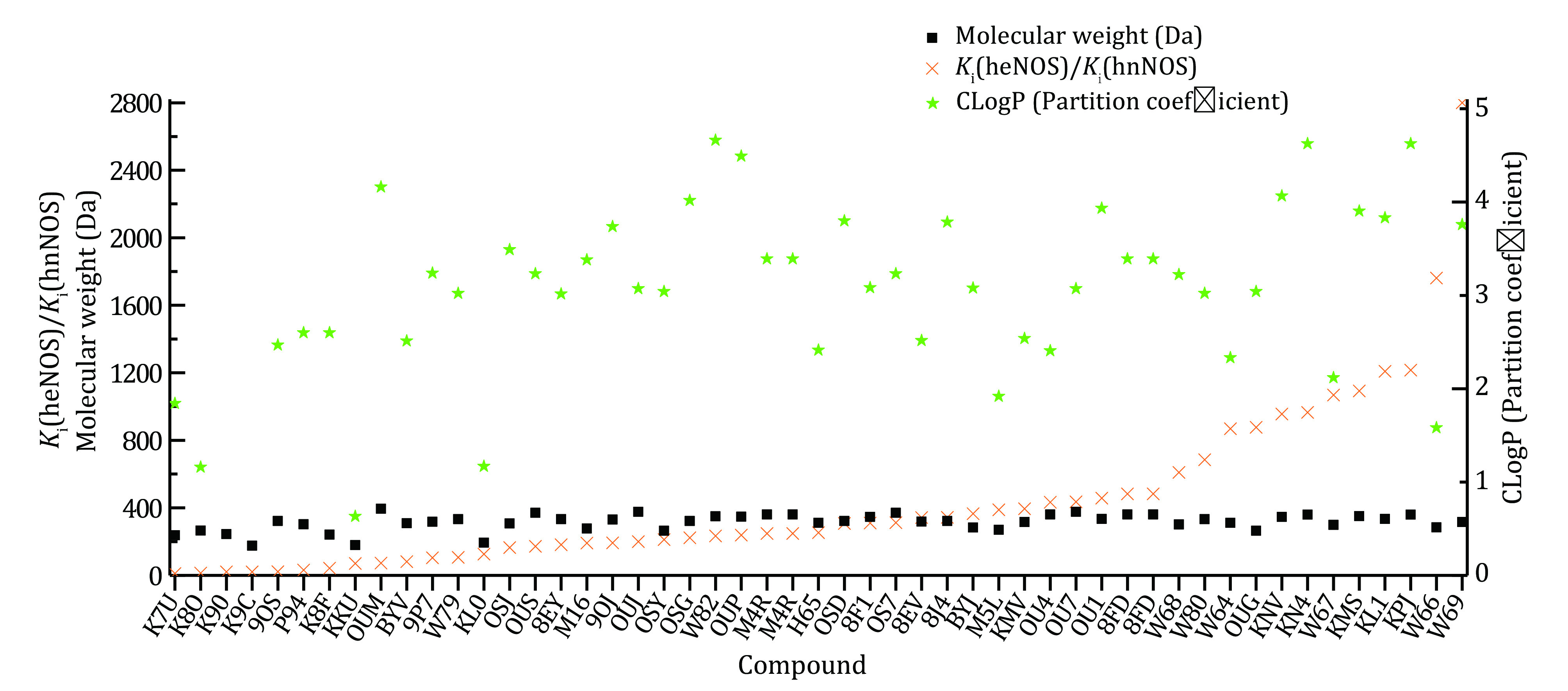
Compounds’ NOS isoform selectivity over other isoforms is neither linked to compound size nor linked to compounds’ hydrophobicity or hydrophilicity. The orange colored cross represents the ratio of experimental *K*_i_ (*K*_i_ value of human eNOS divided by *K*_i_ value of human nNOS)

Statistically, both binding interface area (size) and hydrophobicity (or lipophilicity) of the compound constructively contribute to the energetically favored affinity between human eNOS and compounds, and no such pattern is observed for human nNOS (Fig. 2 and Fig. 3) or iNOS. With the contribution of heme included, this statement still holds. But to target human nNOS or iNOS, selectivity cannot be achieved through a decrease or increase of compound size or alteration of the compound’s hydrophobicity, as selectivity is not linked to hydrophilicity or hydrophobicity of the compound either (Fig. 8). Therefore, although the binding preference of eNOS to compounds do exist, and the different binding profiles between compounds and eNOS, iNOS and nNOS do exist; it is unlikely to actually take advantage of for drug design. The difference in binding features is simply not the way helpful for inhibitor design targeting nNOS and iNOS, or activator design targeting eNOS. A closer look at this issue further explains why.

**Figure 9:**
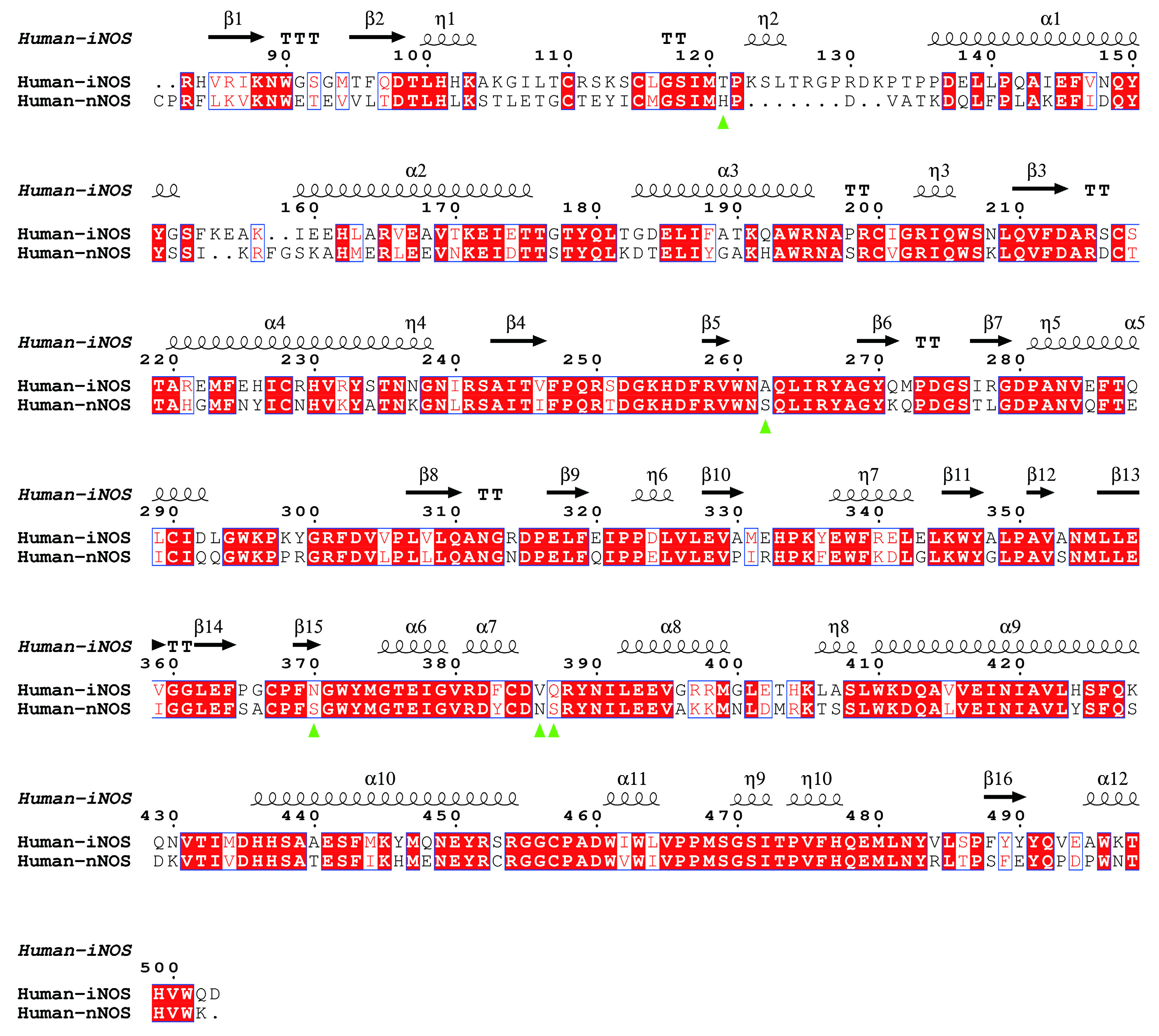
Sequence alignment of human iNOS and human nNOS based on structure superimposition (between iNOS monomer from 3e7g.pdb and nNOS monomer from 6av3.pdb). Human iNOS residues Thr121, Ala262, Asn370, Val386, Gln387, which correspond to human nNOS His342, Ser482, Ser590, Asn606 and Ser607, are highlighted by green triangles. These residues are located at the entrance of the substrate (or inhibitor) binding pocket. RMSD between human iNOS (3e7g.pdb) and human nNOS (6av3.pdb) monomers is ~0.63 Å

**Figure 10:**
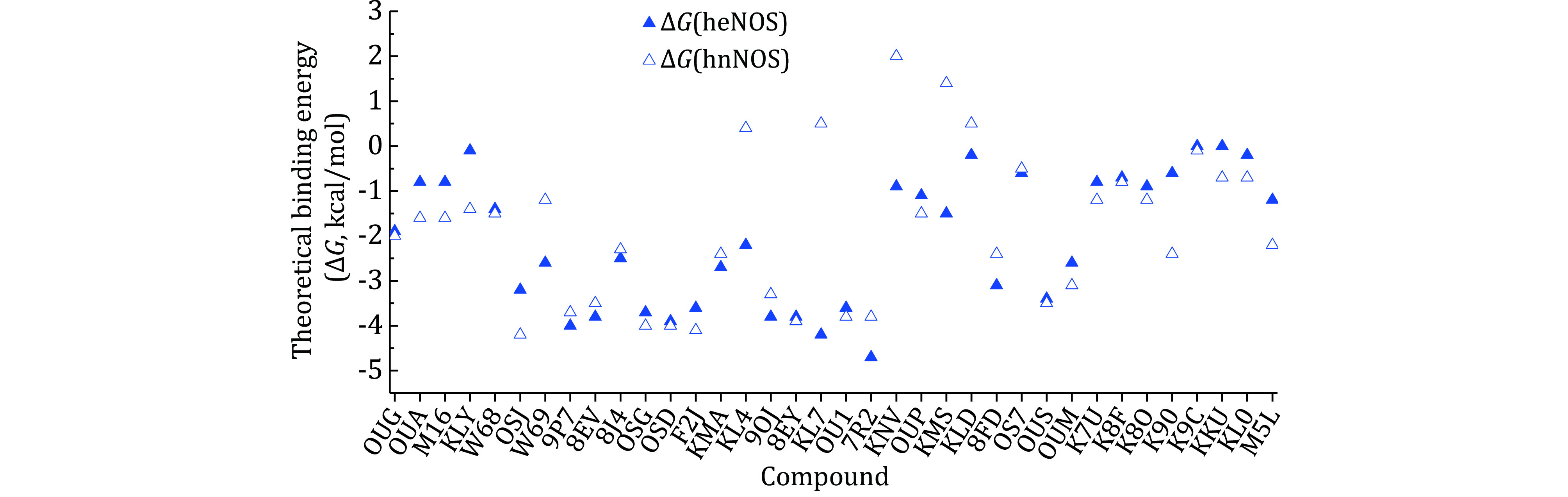
Scatter plot of compounds’ theoretical standard binding free energies (Δ*G*) to human eNOS and human nNOS

Nearly all inhibitors interact with heme b and residues Pro570, Val572, Phe589, Ser590 (back bone), Trp 592, Glu597 of nNOS (Fig. 4), and Pro334, Val336, Phe353, Ser354 (back bone), Trp356, Glu361 of human eNOS (Fig. 4) and Pro350, Val352, Phe369, Asn370 (back bone), Trp372, Glu377 of iNOS (take AT2 (AR-C95791) for example, supplementary Fig. S2, 3E7G.pdb), respectively, and these residues correspond to one another in structure superposition (Fig. 6 and Fig. 9). These residues are highly conserved among human NOS isoforms. Structural analysis of human eNOS, nNOS and iNOS, and structure-based sequence alignment show that the major difference at active sites of human NOS isoforms is residues Asn366 of eNOS, corresponding to Asp602 of nNOS and Asp382 of human iNOS (Fig. 6 and Fig. 9). Only a couple of inhibitors take this advantage successfully (Cinelli *et al.* 2020b). And the conformation of the certain compound is not the same if it binds to different isoforms of human NOS (take for example KL4, human nNOS (6ng6.pdb) and eNOS (6nh3.pdb); KNV, eNOS (6nhf.pdb) and nNOS (6nhc.pdb); KMS, eNOS(6nh8.pdb) and nNOS (6ngd.pdb), supplementary Figs. S3, S4 and S5), making it very difficult to evaluate compound-NOS interactions through computational prediction, and imposing extra complications on rational drug design. Besides the inhibition constant (*K*_i_) of inhibitors to indicate the effect and efficacy at the molecular level (Fig. 8), another parameter, the theoretical binding free energy (Δ*G*) obtained from structural analysis, is used here to analyze the compounds in detail (Fig. 10). *K*_i_ is an experimentally obtained parameter, while Δ*G* value reveals the combined overall interaction profile between compound and target protein. For some compounds, the conformation remains virtually the same when bound to human eNOS and human nNOS, and the theoretical binding energy Δ*G* values are close to each other, too, for example, 8EV (supplementary Fig. S6). Not all large conformational differences of compounds result in significant binding free energy differences to human eNOS and human nNOS (for example, compound KLD, supplementary Fig. S7), and sometimes virtually the same conformation can result in obvious binding energy difference (Take KL7 for example, it is because different residues are involved, Val104 and Phe105 of eNOS and Met341 and His342 of nNOS, supplementary Fig. S8). All these issues lead to a situation in which only after structure determination and experimental examination can the compounds’ activity, efficacy, selectivity and corresponding mechanism be ascertained. Further increase of selectivity via rational improvement seems to be out of reach, and the selectivity relies a lot on chance. Molecular enzymology and structural pharmacology can be quite helpful in drug discovery (Stuehr and Haque 2019).

The results revealed that the binding interface area and binding energy of some intended compounds are inconsistent. This discrepancy can be explained here and may explain why this active site of human NOS may not be the best choice for drug discovery. The drug optimization process often aims for an improved Δ*G*, and there are actually two independent contributors of the Δ*G* value, the enthalpy (Δ*H*) and the entropy (Δ*S*). Some compounds are mainly entropically driven binding, and some others are mainly enthalpically driven binding. There may be compensation or cancellation of the enthalpy (Δ*H*) and the entropy (Δ*S*) in actual optimization practices. A stronger enthalpic binding is usually achieved after rounds of optimisations for a compound to be at the best of its class. To optimize ligands for more enthalpic binding has become the current trend, although the compound that is the first of its class may be dominated by the entropic contribution.

Interference of H4B binding is another source of inhibition of human NOS activities by certain current inhibitors. H4B is a natural cofactor and stabilizer of eNOS; the presence of H4B will stabilize the human NOS dimer, and increase the accumulated functional protein level and overall NO production amount. Without H4B, eNOS may become uncoupled and cannot function properly, while the presence of H4B stabilizes human eNOS both *in vitro* and *in vivo* (Rodriguez-Crespo *et al.* 1997); the addition of H4B to the cultured cell or experimental animal elevates accumulated eNOS protein level and biological NO amount and improves endothelial dysfunction, hypertensive diastolic dysfunction, ischemia-reperfusion injury, myocardial infarction and diabetic nephropathy conditions (Faria *et al.* 2012; Roe and Ren 2012). The elevated NO generation in response to H4B addition probably results from the accumulation of active eNOS enzymes, and the eNOS expression is probably not modified. And 7-nitroindazole (7NI), which competes with the H4B cofactor for the H4B binding site, inhibits dimerization of the nNOS enzyme, and this can lead to pancreatic β-cell dysfunction (Lajoix *et al.* 2004). Consistent with discoveries from this study, H4B was found to play an important role in human NOS catalytic activity as well (Adak *et al.* 2000; Ost and Daff 2005; Wei *et al.* 2003, 2005).

In summary, the two sites, the active site and the H4B binding pocket, currently employed in inhibitor design targeting human nNOS or iNOS, are not competent enough. The very high similarity between oxygenase domains of these three human NOS isoforms in both catalytic function and three-dimensional structure, especially in the physical, and chemical features of the two sites, makes the two strategies very difficult to further increase selectivity and efficacy over other isoforms in drug discovery. Appreciating these facts, alternative strategies are suggested here. With 59% overall polypeptide sequence identity between full-length human eNOS and nNOS, and 53.6% primary amino acid sequence identity between full-length human iNOS and nNOS, and 50% sequence identity between full-length human iNOS and eNOS, the three isoforms are highly similar to each other, with some differences at the N terminal and other regions like the auto-inhibitory loop, *etc*. For nNOS, the additional N-terminal PDZ domain (Fig. 1), which is highly conserved among mammals (supplementary Fig. S9), may be targeted. For human iNOS, which lacks the auto-inhibitory element within the FMN binding region (Fig. 1), the fine exceptional structural features at this region may be exploited.

The plasma concentration of L-arginine is around 200 μmol/L in human bodies (Greene *et al.* 2013), while the maximum physiological concentrations of nitric oxide are around 1–5 μmol/L, and the damaging effect of NO as a free radical at higher concentrations has been widely recognized (Murphy 1999; Weinberger *et al.* 2001). Therefore, the activity of NOS needs to be elegantly moderated. Multidisciplinary effort needs to be endeavored to overcome the obstacles caused by strong conservation among three NOS isozymes, to obtain compounds that discriminate between nNOS, eNOS and iNOS, and to relieve the symptoms of corresponding disease. As naturally existing compounds in the human body, targeted supplements of H4B, arginine or agmatine may be one of the best ways to stabilize eNOS, increase NO bioavailability and achieve vasodilatory, anti-inflammatory, antithrombotic, antiproliferative and cardiovascular protective effects. Structure-activity-relationship analysis is usually the major part of conventional medicinal design, and if the properties of the drug target are taken into consideration, a larger picture will be shown. Based on the discoveries here, preliminary research of natural compounds from traditional Chinese medicine has shown that, the cardiovascular effect of norcoclaurine on mankind probably originated from its interaction or direct relation with human NOS proteins, especially eNOS (Lee *et al.* 2013; Wen *et al.* 2021; Zhu *et al.* 2022). This work provides significant fresh insights into structural and medicinal research on human NOS and bound compounds. Additionally, this research provides a fresh way of comprehensive analysis for drug discovery or dietary supplement research.

## MATERIAL AND METHODS

Structures of human eNOS (Aoyagi *et al.* 2003; Cinelli *et al.* 2020b, 2017; Do *et al.* 2017, 2019; Fischmann *et al.* 1999; Furukawa *et al.* 2017; Garcin *et al.* 2008; Li *et al.* 2014b, 2018; Pensa *et al.* 2017; Piazza *et al.* 2012, 2014, 2016; Rosenfeld *et al.* 2002), nNOS (Alderton *et al.* 2001; Aquilano *et al.* 2008; Cinelli *et al.* 2020b, 2015, 2017; Do *et al.* 2017, 2019; Erdal *et al.* 2005; Hall *et al.* 1994; Huang and Silverman 2013; Kang *et al.* 2015; Li *et al.* 2018; Mukherjee *et al.* 2015; Pensa *et al.* 2017; Silverman 2009; Tang *et al.* 2015; Wang *et al.* 2016), and iNOS (Fischmann *et al.* 1999; Garcin *et al.* 2008; Li *et al.* 2014a) as of June 9^th^ 2023 are downloaded from Protein Data Bank. Binding interface area, theoretical binding free energy (Δ*G*) between compounds and eNOS, nNOS are obtained by using EBI webserver PDBePisa (Krissinel and Henrick 2007). Although binding interface area and theoretical binding free energy between compounds and heme are significant and can not be neglected, because the same heme moiety from the three NOS isoforms will not contribute to the selectivity and specificity of the compounds, and to include the contribution of heme would be quite another way of analysis (supplementary Figs. S10–S12 and supplementary Tables S6–S7), therefore, the second way of analysis (with the contribution of heme included) is performed separately. On one side, the compound binds to the polypeptide chain, and the binding interface area and theoretical binding free energy between the compound and polypeptide chain are summarized in the supplementary Tables S2 and S3. On the other side, the compound binds to the heme, and the binding interface area and theoretical binding free energy between the compound and heme are summarized in the supplementary Tables S6 and S7. On the other hand, the slight difference in the binding pocket imposed by different NOS isoforms may affect the orientation of the compound in a way that the binding with even the same heme moiety (from different NOS isoforms) may differ somehow. To examine the relationship between the sum of these interactions and specificity, they are summarized and discussed in detail in the supplementary file (supplementary Figs, S10–S12). ClogP values (Mol Refractivity and Partition Coefficient) are obtained from computations by ChemBio 3D. RMSDs (root mean square deviation) between different eNOS structures are obtained through alignment and superposition by using UCSF Chimera (Pettersen *et al.* 2004). Detailed summary, comparison and original statistics are shown in the supplementary Tables S1–S7. All the analyzed eNOS and nNOS structures are homo dimers or tetramers, and there are at least two identical compounds in each of the published eNOS or nNOS structures, with at least one compound bound to each monomer. The reported interface area and Δ*G* values in the figures here are averages of that of the protein monomers. The volume (or size) and the shape of the substrate binding pockets of human NOS isoforms are worked out by using POCASA 1.1 (Yu *et al.* 2010). POCASA 1.1 parameters used in this study are default values, the Probe Radius is set to 2 Å, and the grid size is 1 Å. The single point flag is 16, and the protein depth flag is 18. Human nNOS K301R, R354A and G357D mutation sites are far away from the pockets discussed here, and the overall structure of the protein or the issues discussed here are not affected by these mutations, therefore, the mutant structures are not excluded from analysis and mutation issues not raised in discussion. Full-length NOS structures are modified from alpha fold predictions (Jumper *et al.* 2021). Cartoon and other structure model representations are generated by using UCSF chimera (Pettersen *et al.* 2004) or PyMOL (http://www.pymol.org). Several parameters are considered here to be medicinally helpful, from the compound’s point of view, its molecular weight, size or volume, structure, shape, hydrophilicity, polarity, charge, *etc.*; from the drug target or the interaction point of view, the binding position, pocket size, shape, hydrophobicity, polarity, charge property of the pocket, residue composition, compound’s binding conformation, interaction profile, the covalent or non-covalent interactions involved, *etc*.

## Conflict of interest

Jianshu Dong, Dié Li, Lei Kang, Chenbing Luo and Jiangyun Wang declare that they have no conflict of interest.
